# Targeted anti-angiogenesis therapy for advanced osteosarcoma

**DOI:** 10.3389/fonc.2024.1413213

**Published:** 2024-08-26

**Authors:** Qiao Zhang, Yuxuan Xia, LiYuan Wang, Yang Wang, Yixi Bao, Guo-sheng Zhao

**Affiliations:** ^1^ Department of Pain and Rehabilitation, Xinqiao Hospital, Army Medical University, Chongqing, China; ^2^ Department of Clinical Laboratory, The Second Affiliated Hospital of Chongqing Medical University, Chongqing, China; ^3^ Department of Spine Surgery, The Second Affiliated Hospital of Chongqing Medical University, Chongqing, China; ^4^ Department of Emergency Medicine Center, Sichuan Provincial People’s Hospital, University of Electronic Science and Technology of China, Chengdu, China

**Keywords:** advanced osteosarcoma, anti-angiogenesis therapy, mechanism of tumor angiogenesis, proangiogenic factors, comprehensive therapy

## Abstract

To date, despite extensive research, the prognosis of advanced osteosarcoma has not improved significantly. Thus, patients experience a reduced survival rate, suggesting that a reevaluation of current treatment strategies is required. Recently, in addition to routine surgery, chemotherapy and radiotherapy, researchers have explored more effective and safer treatments, including targeted therapy, immunotherapy, anti-angiogenesis therapy, metabolic targets therapy, and nanomedicine therapy. The tumorigenesis and development of osteosarcoma is closely related to angiogenesis. Thus, anti-angiogenesis therapy is crucial to treat osteosarcoma; however, recent clinical trials found that it has insufficient efficacy. To solve this problem, the causes of treatment failure and improve treatment strategies should be investigated. This review focuses on summarizing the pathophysiological mechanisms of angiogenesis in osteosarcoma and recent advances in anti-angiogenesis treatment of osteosarcoma. We also discuss some clinical studies, with the aim of providing new ideas to improve treatment strategies for osteosarcoma and the prognosis of patients.

## 1 Introduction

Osteosarcoma is a bone-derived primary malignant tumor that is characterized by malignant proliferation and the production of bone-like tissue and matrix (1, 2). Epidemiological investigations showed that the age distribution of patients with osteosarcoma was bimodal. It mainly occurs in children and young adults with rapid bone growth between 10 and 30 years old, and in certain people over 65 years old (3–5). The first peak group coincides with the peak of adolescent growth, and the second is thought to be secondary to long-term Paget’s disease and radiation therapy (6–9). Osteosarcoma is considered to be concealed, malignant, and aggressive. Approximately 25% of patients with osteosarcoma present with metastasis at the time of initial diagnosis and experience recurrence during the treatment (10, 11). Osteosarcoma mainly occurs at the epiphyseal end of the long leg bone, for example, around the knee and near the humerus. Patients often experience pain, swelling, restricted limb activity, and accessible clumps at the lesion site (2). Diagnosis depends mainly on pathological biopsy. Osteosarcoma can be classified into eight categories according to fifth edition of the 2020 World Health Organization (WHO) classification of tumors of soft tissue and bone tumors, including low-grade central osteosarcoma, conventional osteosarcoma, telangiectatic osteosarcoma, small cell osteosarcoma, parosteal osteosarcoma, periosteal osteosarcoma, high-grade surface osteosarcoma, and secondary osteosarcoma (12). According to the degree of malignancy, osteosarcoma can be classified into low and high grade. Low-grade osteosarcoma, which consists of low-grade central osteosarcoma and cortical osteosarcoma, is less malignant, and can usually be treated using surgery alone. However, high-grade osteosarcoma is one of the most malignant tumors, and is often accompanied by lung metastases. High-grade osteosarcoma always requires surgery combined with neoadjuvant chemotherapy and postoperative chemotherapy, frequently requiring other comprehensive therapies. Unfortunately, the prognosis of patients with osteosarcoma has not improved significantly after nearly 40 years of treatment using the multi-mode combination strategy of surgical and neoadjuvant chemotherapy or postoperative chemotherapy (13–15). According to statistics, the 5-year survival rate of primary osteosarcoma can reach about 65–70% (16, 17). However, for advanced osteosarcoma, which almost always involves lung metastasis, the 5-year survival rate is only approximately 20% (18–21). It is believed that high invasiveness and resistance are the main causes of poor treatment efficacy; therefore, new treatment strategies are urgently required (22–24).

Tumor growth and progression are intricately linked to angiogenesis, which is essential for the provision of nutrients. Moreover, the formation of new blood vessels facilitates the metastatic dissemination of cancer cells into circulation and subsequent establishment of metastases (3, 20). Thus, anti-angiogenesis treatment has become an important therapeutic strategy and comprehensive therapy for advanced tumors, aiming to limit the growth and metastasis of tumors by inhibiting neoangiogenesis and normalizing tumor vessels (25). For example, the classic anti-angiogenesis drug, Bevacizumab, has been approved to treat lung cancer, hepatocellular carcinoma, glioma, and colorectal cancer, and has demonstrated desirable clinical efficacy (26).

Advanced osteosarcoma, namely with metastatic, recurrent or unresectable diseases, is a highly vascularized tumor, and the progression of advanced osteosarcoma is closely related to angiogenesis (27). Angiogenesis not only plays a pivotal role in the metastasis of osteosarcoma, but also facilitates the colonization of tumor cells at secondary sites (1, 3). Therefore, inhibiting angiogenesis appears to be beneficial for preventing advanced progression of osteosarcoma. Additionally, studies have shown that the microvascular density of osteosarcoma correlates positively with tumor prognosis, and the expression of vascular endothelial growth factor (VEGF), induced by angiogenesis, has been used as an important method to evaluate the prognosis of osteosarcoma (20). Although anti-angiogenesis therapy represents a promising strategy to treat advanced osteosarcoma, currently, the therapeutic effect of anti-angiogenesis treatment in osteosarcoma remains controversial (25). Thus, this review aims to summarize the molecular mechanism of angiogenesis and the current role of anti-angiogenesis therapy in advanced osteosarcoma to provide new ideas for its effective treatment.

## 2 The mechanism of angiogenesis in osteosarcoma

Angiogenesis is a complex and dynamic process that is regulated by the homeostasis of angiogenic and anti-angiogenic factors in the physiological state (28, 29). In the tumor microenvironment (TME), angiogenic homeostasis is disrupted, resulting in excessive angiogenesis in the lesion (30), which provides nutritional support for the tumor, thereby promoting the growth, invasion, and metastasis of osteosarcoma (31, 32). The expansion of osteosarcoma relies on neovascularization to maintain oxygen and nutrient supplies (33), and the rapid growth of osteosarcoma subsequently opens the “angiogenesis switch” caused by the higher oxygen demand in the high metabolic TME, forming a vicious circle. Large amounts of neoangiogenesis lead to disordered tumor vessels, neovascular dysfunction, and low perfusion within the TME (34), thus promoting the growth, invasion, immunosuppression, and distant metastasis of osteosarcoma (35).

### 2.1 Angiogenesis patterns in tumors

**Figure 1:**
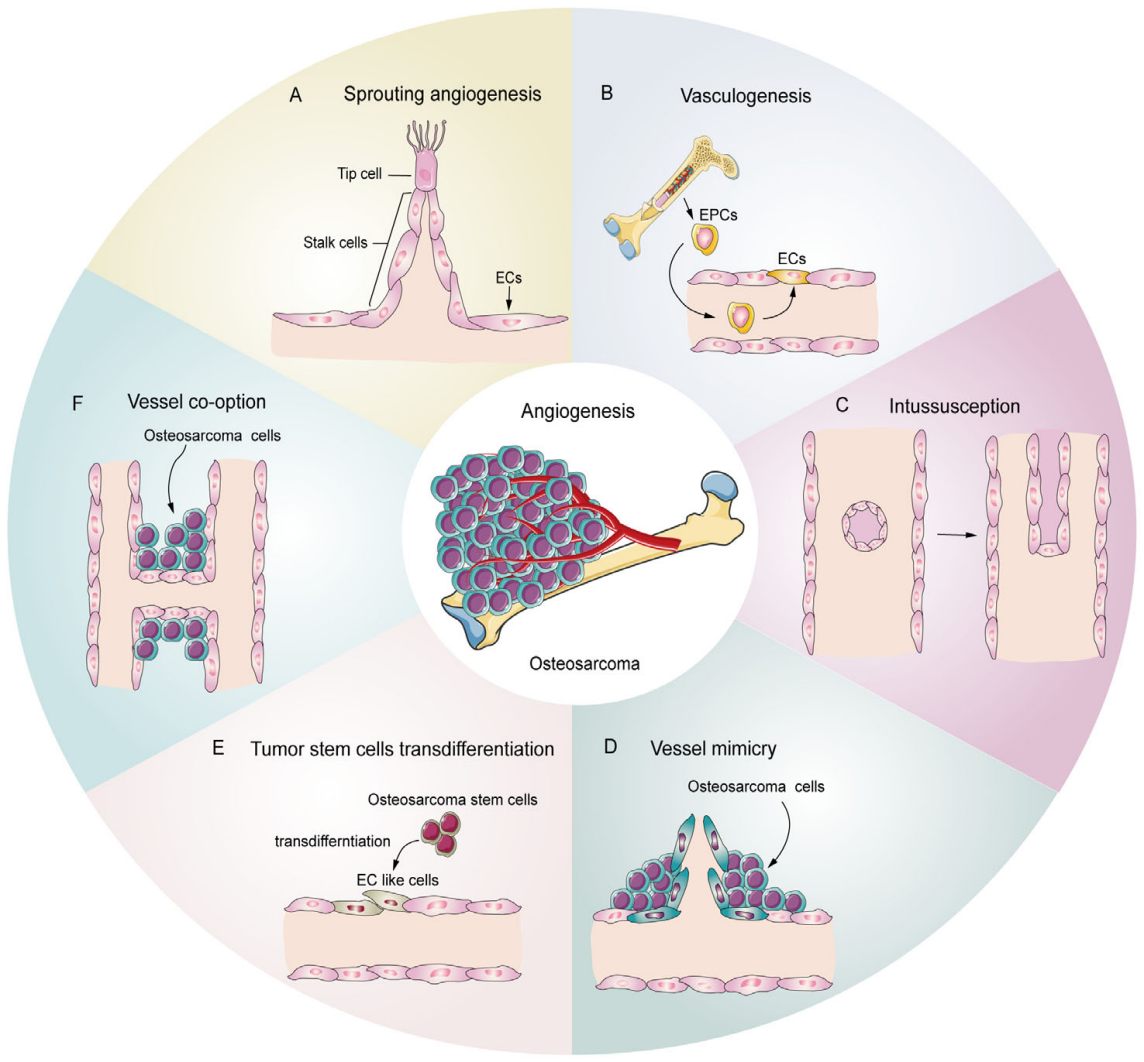
Potential schematic diagram of osteosarcoma angiogenesis patterns. **(A)** Sprouting angiogenesis: the main pattern in osteosarcoma, which is induced by tip cells and stalk cells. **(B)** Vasculogenesis: EPCs are recruited and differentiate into ECs to participate in angiogenesis **(C)** Intussusception: an existing vessel is split into two vessels through EC reorganization. **(D)** Vessel mimicry: tumor cells form tubular structures to sustain tumor perfusion. **(E)** Tumor stem cell transdifferentiation: osteosarcoma stem cells differentiate into EC like cells and participate in angiogenesis. **(F)** Vessel co-option: osteosarcoma cells colonize around the existing vessels.

Tumors, including osteosarcoma, demonstrate several patterns of angiogenesis (**Figure 1**), such as sprouting angiogenesis (36, 37), intussusception angiogenesis (38), vasculogenesis (39), vessel mimicry (40, 41), trans-differentiation of tumor stem cells (26, 42, 43), and vessel co-option (44). Sprouting angiogenesis involves the formation of a sprouting bud based on existing blood vessels and is the main pattern of physiological and pathological angiogenesis (45) (**Figure 1A**). Under hypoxia, quiescent endothelial cells (ECs) are activated by angiogenic stimulators, such as VEGF, and are converted into tip cells or stalk cells. Tip cells are characterized by their location at the tip of the sprouting buds, which contain many filopodia to sense the VEGF concentration gradient. They guide the direction of angiogenesis, but lack proliferative activity (46). Stalk cells are located behind the tip cells. They are highly proliferative and mainly form the lumen to elongate the vascular buds under tip cell guidance (47). Vasculogenesis is the process by which the bone marrow-derived endothelial progenitor cells (EPCs) differentiate into ECs and migrate inside the tumor to form new vessels (48) (**Figure 1B**). Intussusception angiogenesis is a process that inserts mesenchymal structures into the interior of a pre-existing vessel, splitting the vessel into two vessels, which is considered an important complementary modality to sprouting angiogenesis (49, 50) (**Figure 1C**). Vessel mimicry involves the formation of a “microvascular channel” by EC-like tumor cells with the extracellular matrix (ECM), which exposes tumor stem cells to the blood stream, thereby facilitating metastasis (40, 51) (**Figure 1D**). Tumor stem cells can participate in angiogenesis inside tumors by transforming into EC-like cells (52) (**Figure 1E**). Finally, vessel co-option is not a true angiogenesis, but comprises a pattern in which tumor cells colonize around existing blood vessels to encapsulate them inside the tumor (44) (**Figure 1F**).

### 2.2 Molecular mechanism of angiogenesis

**Figure 2:**
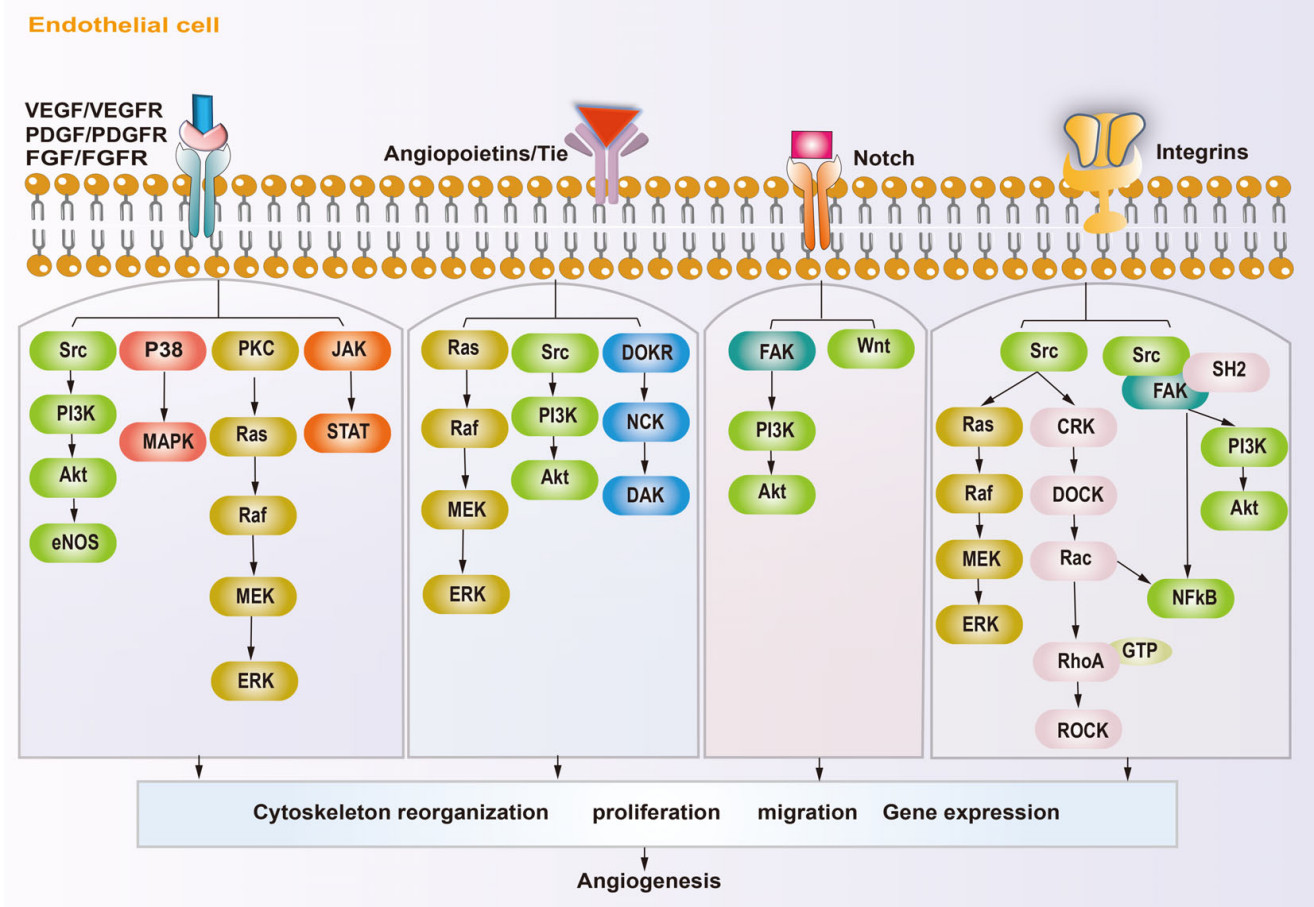
Schematic diagram showing the angiogenic mechanism of osteosarcoma. Angiogenesis-promoting factors interact with receptors on endothelial cell, triggering specific signaling pathways, subsequently reorganization cytoskeleton, proliferation, migration and gene expression, ultimately influence angiogenesis.

In osteosarcoma, the fast-growing tumor and the lagging angiogenesis result in a long-term hypoxic TME. The main targets affecting angiogenesis are VEGF, hypoxia inducible factors (HIFs), platelet-derived growth factor (PDGF), epidermal growth factor (EGF), fibroblast growth factor (FGF), hepatocyte growth factor (HGF), insulin like growth factor (IGF), transforming growth factor-β (TGF-β), and angiopoietins (ANGs) (53, 54). HIFs accumulate inside the tumor during hypoxia and subsequently cause high expression of VEGF, which rapidly stimulates ECs (55, 56). The VEGF family includes VEGF-A\B\C\D\E\F and placental growth factor (PLGF). VEGF-A and vascular endothelial growth factor receptor 2 (VEGFR2) are the main inducers of angiogenesis and the major targets of anti-angiogenesis therapy. VEGF-A activates downstream phosphatidylinositol-4,5-bisphosphate 3-kinase (PI3K)/protein kinase B (AKT), P38, and extracellular regulated kinase (ERK)/mitogen activated protein kinase (MAPK) pathways after binding with VEGFR2, which then promote the proliferation and migration of ECs, resulting in angiogenesis (57–59). In addition, studies have shown that growth factors, such as FGF and PDGF, also promote tumor angiogenesis through VEGF/VEGFR pathways (41, 43, 60). PDGF, TGF-β, angiopoietins/TEK receptor tyrosine kinase (TIE) are also active in the maturation of new blood vessels. ECs attract PDGFR-β+ pericytes to complete the vascular barrier by releasing PDGF-β. Suppressing PDGFR-β signals can lead to a decrease in pericellular coverage and pericellular detachment, resulting in vascular dysfunction and inhibition of tumor growth (61). In the physiological state, angiopoietin 1 (ANG-1) is mainly expressed around ECs, where it activates TIE-2 to mediate vascular maturation. ANG-2 is released by tip cells and mainly has anti-ANG-1 functions, guiding vascular degeneration (62, 63). In the TME, the abundant tip cells secrete excess ANG-2, resulting in disturbance of ANG-1 and ANG-2 homeostasis, thus leading to immature neovascularization (30). The Notch and Wnt signaling pathways also participate in tumor angiogenesis. The Notch signaling pathway is involved in the dynamic regulation of tip cells and stalk cells. Suppression of Notch signaling can lead to a tip cell phenotype (47), and activating Notch signaling leads to a stalk cell phenotype and activates the Wnt pathway, which facilitates the proliferation of the stalk cell phenotype (42), thus promoting vascular bud formation. Integrins are transmembrane receptors that mediate adhesion between cells and extracellular matrices. They promote growth factors like VEGF and FGFs, and enhance ANG-1 binding with its receptors VEGFR-2 and FGFRs. Integrins can also promote the maturation of neovascular tissue and regulate the connection of ECs with the ECM (64). The molecular mechanism of angiogenesis in osteosarcoma is summarized in **Figure 2**.

### 2.3 Other potential proangiogenic factors in osteosarcoma

There are some other potential proangiogenic factors and cytokines that participate in osteosarcoma angiogenesis, which could be promising targets for anti-angiogenesis therapy in osteosarcoma. Human interleukin (IL) family members have been shown to play important roles in regulating the immune and inflammatory responses (65, 66). Recently, several IL family members were found to participate in the regulation of the angiogenesis in osteosarcoma. Tzeng et al. reported that IL-6 could upregulate VEGF expression through the apoptosis signal-regulating kinase 1 (ASK1) and P38 pathways, and induced angiogenesis in osteosarcoma (67). IL-34 is associated with the progression of osteosarcoma and an increase in neo-angiogenesis (68). Moreover, some evidence suggests that IL-1 and IL-8 also show certain proangiogenic effects in osteosarcoma (69–71). In addition, the expression of IL-17A, IL-1β and IL-10 was proven to be involved in osteosarcoma carcinogenesis (72–74). Moreover, the latest research indicates that IL-17A, IL-1β, and IL-10 might be related to angiogenesis in other types of cancer (75–77). Thus, targeting them might be a promising strategy to induce anti-angiogenesis effects in osteosarcoma.

Tumor necrosis factor alpha (TNF-α) is involved in many tumor cell pathological cellular pathways, including tumor invasion, epithelial-mesenchymal transition, vascular invasion, and the destruction of tumor vasculature (78, 79). Ségaliny et al. reported that TNF-α could stimulate osteosarcoma cells to secrete IL-34 and increased their angiogenesis (68). Moreover, recent evidence suggested that nuclear factor kappa B (NF-κB) and HIF-1α pathways might be potential downstream targets to regulate angiogenesis in tumors (79). However, further study is needed to confirm this molecular mechanism in osteosarcoma.

Endothelial-specific molecule 1 (ESM1) is thought to be a tip cell marker, and was found to be significantly related to angiogenesis (80). Angiopoietin-like proteins (ANGPTLs) are similar to angiopoietin and also promote angiogenesis; however, they do not bind to the TIE family of angiopoietin receptors (81). In recent years, these two types of proteins have gained increasing significance as their proangiogenic effect in cancers have been explored (80, 82, 83). Recently, a study found that ANGPTL2 could enhance angiogenesis in osteosarcoma by upregulating the expression of hexokinase 2 (HK2) and VEGF (81). However, so far, there has been little research on the roles of these proteins in osteosarcoma. A more detailed understanding of how these proteins function in osteosarcoma angiogenesis is required to develop targeted therapy.

### 2.4 Metabolism regulates tumor angiogenesis

Current anti-angiogenic therapy targeting VEGF and its related pathways has not achieved the desired results in osteosarcoma, which has forced scientists to explore new anti-angiogenic strategies (3, 16, 25). Although little research has focused on targeting EC metabolism in the anti-angiogenic therapy of osteosarcoma, it has received increased research attention (84), and might represent an effective way to inhibit osteosarcoma angiogenesis.

**Figure 3:**
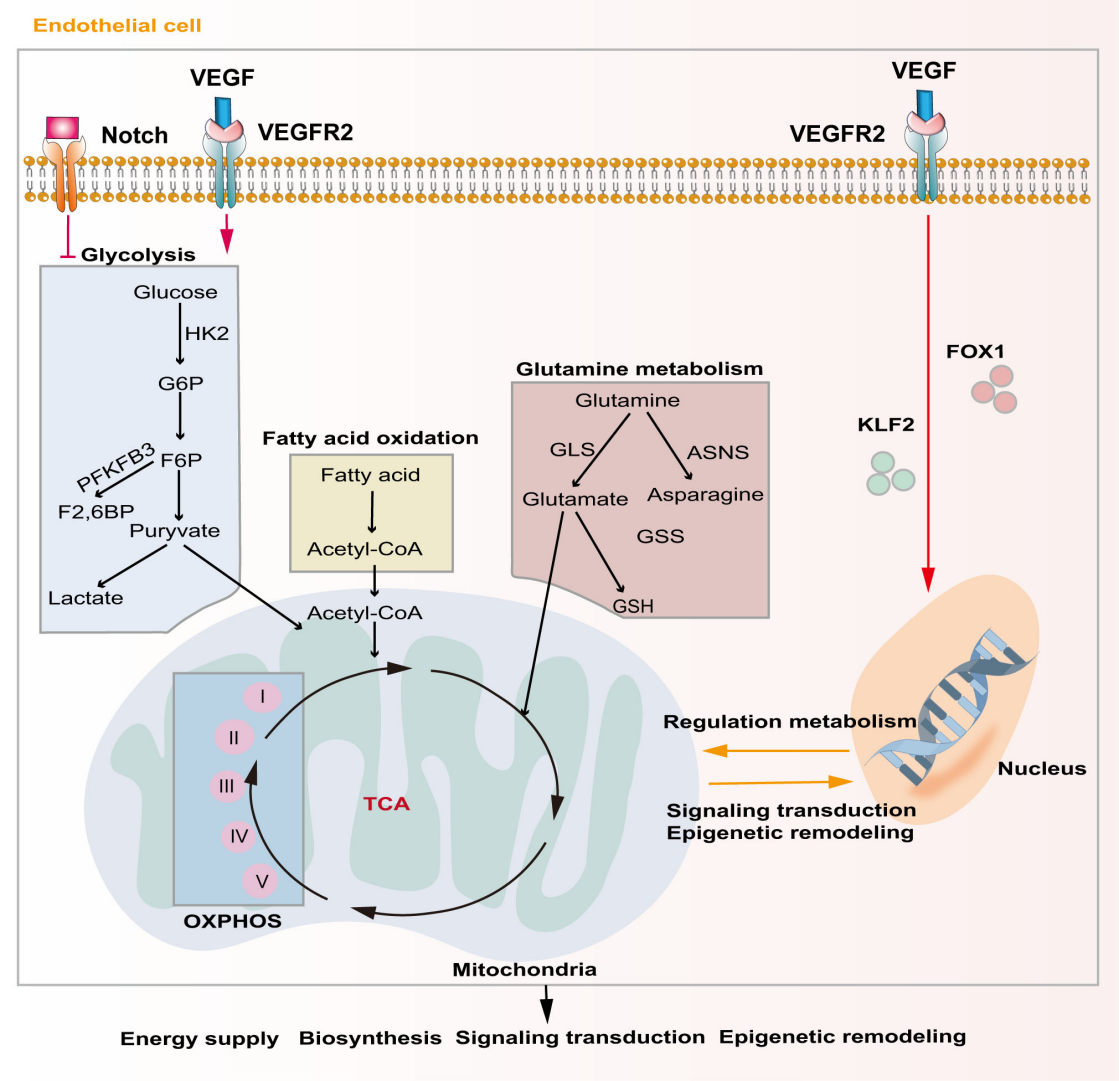
Major metabolic pathways of endothelial cells during angiogenesis. Activation of the Notch signaling pathway inhibits intercellular glycolytic level. VEGF binds to VEGFR2 and enhances downstream glycolysis flux. Additionally, VEGF regulates gene expression and metabolism in endothelial cells via activating of FOXO1 and KLF2. Metabolic products like acetyl-CoA and glutamate act in energy supply, biosynthesis, signal transduction and epigenetic remodeling through mitochondrial-related oxidative phosphorylation pathways.

Unique metabolic fluxes and metabolic patterns are closely related to survival, phenotypic transformation, migration, and proliferation. Studies have shown that EC metabolic reprogramming regulates angiogenesis through energy supply, biosynthesis, signaling transduction, and epigenetic remodeling (**Figure 3**). Unlike other cells, ECs rely mainly on aerobic glycolysis for energy, although they are directly exposed to the hyperoxic, high-sugar blood environment, which is similar to the Warburg effect of cancer cells (85, 86). In the physiological state, phalanx ECs attach to the intravascular surface in a hibernation state, maintaining the endothelial barrier via a low aerobic glycolysis flux. When ECs are awakened by angiogenic factors, the transcription factors forkhead box O1 (FOXO1) and Kruppel-like factor 2 (KLF2) are activated, and the quiescent cells transform into tip cells or stalk cells, which is accompanied by a change in cell metabolic patterns (87, 88). ECs obtain a tip cell phenotype via high expression VEGFR2. VEGF upregulates the expression of fructose 6-phosphate-2-kinase/fructose-26-diphosphatase 3 (PFKFB3) and HK2 inside tip cells by binding to VEGFR2, and then increases internal glycolysis levels to meet the high energy demands of tip cells during pathfinding (89). At this point, tip cells activate the Notch signaling pathway in adjacent ECs through lateral inhibition mechanisms, which forces the neighboring cells to transform into stalk cells (47). At the same time, activated Notch downregulates PFKFB3 expression to reduce glycolysis levels, followed by activation of mitochondrial fatty acid oxidation, which promotes the synthesis of nucleotides, endowing the stalk cells with a high proliferation status, thus facilitating angiogenesis (90). In addition to promoting angiogenesis in ECs, PFKFB3 and HK2 are also crucially involved in the progression of osteosarcoma by regulating aerobic glycolysis (91, 92). Therefore, targeting glycolytic enzymes PFKFB3 and HK2 might be a promising therapy for osteosarcoma.

Although ECs mainly rely on glycolysis to maintain their physiological activity, mitochondria also play a crucial role in ECs. Mitochondria-related oxidative phosphorylation (OXPHOS) plays an indispensable role in providing substrates and maintaining the NAD+/NADH ratio in ECs (93). Tricarboxylic acid cycle (TCA)-related products are important substrates for EC biosynthesis and mitochondrial complex I is extremely important for EC proliferation in tumors (94). Indeed, blocking mitochondrial complexes I and III inhibits the proliferation of ECs and causes pathological angiogenesis (95).

Lipid metabolism is also essential to maintain EC structure and function. During angiogenesis, VEGF-B promotes the uptake of fatty acids by upregulating the expression of fatty acid transport protein 3 (FATP3) and fatty acid transport protein 4 (FATP4) (96). Inhibiting fatty acid synthesis reduces the proliferation and migration of ECs by preventing post-translational modification of proteins and the mechanistic target of rapamycin kinase (mTOR) pathways, ultimately resulting in sprouting angiogenesis (97). Fatty acid oxidation (FAO) maintains EC proliferation by increasing the synthesis of aspartate through mitochondria (98). Another major substrate of mitochondrial respiration in ECs is glutamine, which plays important roles not only in biosynthesis, but also in replenishing the TCA cycle as a carbon source (99). Moreover, the integrity of glutamine metabolic pathways is closely related to the migration of tip cells and the proliferation of stalk cells. Glutathione, a downstream product of glutamine, also helps to maintain EC redox homeostasis. Blocking glutamine metabolism pathways by silencing *ASNS* (encoding asparagine synthase) inhibited angiogenesis by stopping tip cell migration and stalk cell proliferation, resulting in suppression of sprouting angiogenesis (100). Recent evidence shows that dihydroartemisinin interferes with lipid metabolism in osteosarcoma cells, especially the FAO process, and impedes antiangiogenic drug resistance (101). However, there is still lack of studies exploring EC lipid metabolism in osteosarcoma.

## 3 Anti-angiogenesis therapy in osteosarcoma

The treatment of osteosarcoma mainly relies on surgery and chemotherapy. As an effective supplement to routine treatment, recently, anti-angiogenesis therapy has received increased attention. Anti-angiogenesis therapy aims to block the nutrient supply of tumors and impede the TME in the following ways: destruction of tumor blood vessels by inducing apoptosis (102); inhibiting angiogenesis by limiting cell proliferation and migration, and promoting the normalization of blood vessels in tumors (103–105); and restoring the perfusion inside tumors (106), thereby altering the TME (43, 89, 90, 107). The current anti-angiogenic treatments for osteosarcoma can be classified into the following categories: Monoclonal antibodies (Mabs), tyrosine kinase inhibitors (TKIs), small molecule inhibitors, Chinese herbal medicine, aptamers, and nano-particles (NPs). The targets and mechanisms of these drugs are shown in **Table 1**, and the clinical trials involving these drugs are listed in **Table 2**.

**Table 1:** Summary of pre-clinical studies related to anti-angiogenesis therapy in osteosarcoma.

Class	Drugs	Mechanism	Ref
Mab	Bevacizumab	Anti-VEGF.	(108–110)
	R1507	Anti-IGF-1R.	(111)
TKIs	Sorafenib	Anti-VEGFR, PDGFR, ERK1/2, MCL-1, Ezrin pathways.	(112–114)
	Sunitinib	Anti-VEGFR, PDGFR.	(115)
	Cediranib	Anti-VEGFR2\3, PDGFR.	(116)
	Apatinib	Anti-VEGFR2\STAT3\BCL-2.	(117)
	Pazopanib	Anti-VEGFR, PDGFR, FGFR.	(118)
	Lenvatinib	Anti-VEGFR1-3, FGFR, PDGFR, RET, KIT.	(119)
	Cabozanitib	Anti-VEGFR2, c-MET, c-KIT, FLT-3, AXL, RET.	(120)
	Regorafenib	Anti-VEGFR1-3, Tie2, PDGFR α\β, FGFR1\2.	(120)
Inhibitor	Everolimus	Inhibition of the mTOR pathway.	(121)
Endostain	Endostar	Anti-VEGF.	(122)
CHM	Thymoquinone	Inhibition of NF-κB.	(123)
	Triptolide	Inhibition of HIF-1α, VEGF, Wnt/β-Catenin.	(124)
	Sinomenine	Inhibition ofCD147, VEGF.	(125)
	phyllanthus urinaria	Decrease CD31.	(126)
	calotropis procera	Inhibition CD31, VEGF, TGF-β.	(127)
Metabolic targets therapy	2-DG	Inhibition of ANGPTLs, HK, LDHA, VEGF.	(81, 128)
	Bavachinin	Inhibition of HIF-1α\HK2.	(129)
	Icariside II	Inhibition of HIF-1α \VEGF.	(130)
Aptamer	LC09	Inhibition of VEGF A.	(131)

Mab, Monoclonal antibody; IGF-1R, insulin-like growth factor-1 receptor; TKIs, Tyrosine kinase inhibitor; CHM, Chinese herbal medicine; VEGF, vascular endothelial growth factor; VEGFR, vascular endothelial growth factor receptor; PDGFR, platelet-derived growth factor receptor; ERK, extracellular regulated protein kinase; MCL-1, myeloid cell leukemia-1; STAT3, signal transducer and activator of transcription 3; BCL-2, B-cell lymphoma-2; FGFR, Fibroblast growth factor receptor; RET, Ret proto-oncogene; KIT, KIT proto-oncogene, receptor tyrosine kinase; c-MET, MET proto-oncogene, receptor tyrosine kinase; FLT-3, Fms related receptor tyrosine kinase 3; AXL, AXL receptor tyrosine kinase; Tie-2, TEK receptor tyrosine kinase; mTOR, mechanistic target of rapamycin kinase; NF-κB, nuclear factor kappa B; HIF-1α, hypoxia inducible factor 1 alpha; Akt, protein kinase B; MMP9, matrix metalloproteinase 9; HK, hexokinase; LDHA, lactate dehydrogenase A.

**Table 2:** Summary of clinical trials related to anti-angiogenesis therapy in osteosarcoma.

Drugs	Combination	Clinical Trial	No of patients	Outcomes	Ref
Cediranib	–	I	4	ORR 25%.	(132)
Sorafenib	–	–	4	3/4 SD.	(133)
	–	I	8	2/8 PD; 2/8 SD; 5-year OS 64%.	(134)
	–	II	35	3/35 PR; 12/35 SD; 4-month PFS 46%; m-PFS 4 months; m-OS 7 months.	(135)
	–	–	7	4 month m-PFS 14.3%;DCR 80.0%;m-PFS 51 days;m-OS 119 days.	(136)
	everolimus	II	38	17/38 SD.	(137)
	everolimus	–	14	4 month m-PFS 30.8%; DCR 91.7%; m-PFS 101 days; m-OS 181 days.	(136)
Apatinib	–	II	37	16/37 PR; ORR 43.24%; 4-month PFS 56.76%; m-PFS 4.5 months; m-OS 9.87 months.	(27)
	–	II	27	ORR 25.93%; DCR 66.67%; m-PFS 3.5 months; m-OS 9.5 months.	(138)
	camrelizumab	II	43	6-month PFS 50.9%; ORR 20.9%.	(139)
Pazopanib	–	–	3	3/3PR.	(140)
	–	–	3	3/3CR.	(141)
	–	–	15	1/15 PR; 6/15 SD; m-PFS 6 months; OS 7months.	(142)
Regorafenib	–	I	3	1/3 PR.	(143)
	–	II	26	DCR 64%; PR 8%; SD 17/26; m-PFS 16.4 weeks.	(144)
	–	II	42	3/22 PR; m-PFS 3.6 months; m-OS 11.1 months.	(145)
	–	–	10	4 month m-PFS 60%;DCR 77.8%;m-PFS 167 days; m-OS 411 days.	(136)
Cabozantinib	–	II	42	PR 12%;6 month PFS33%	(146)
Lenvatinib	–	II	31	ORR 6.7%; m-PFS 3months.	(147)
	Etoposide+ifosfamide	I/II	35	4 month -PFS 51%.	(119)
Bevacizumab	MAP	II	31	4 year EFS 57.5 ± 10.0%;5 year OS 83.4 ± 7.8%.	(148)
	TAG	I	8	ORR 63a; DCR88%; 2/8PR; 3/8CR; 2/8SD.	(149)
R1507	–	II	38	PR 2/38; SD 10/38.	(150)

ORR, objective response rate (complete response +partial response); PD, progressive disease; SD, stable disease; PR, partial response; CR, clinical response; DCR, disease control rate; EFS, event-free survival; PFS, progression-free survival; m-PFS, median progression-free survival; OS, overall survival; m-OS, median overall survival; MAP, methotrexate+ doxorubicin+cisplatin; TAG, docetaxel+ bevacizumab+gemcitabine.

### 3.1 Monoclonal antibodies

VEGFA/VEGFR are the main targets for anti-angiogenic therapy; therefore, Mabs targeting VEGF are also widely used to treat solid tumors, especially osteosarcoma. Bevacizumab was the first Mab to be approved as an angiogenesis inhibitor for solid tumors. Its main anti-angiogenic function is binding to all VEGFA subtypes in the circulation and preventing them from activating VEGFR (108). In a pre-clinical study, Zhao et al. reported that intraperitoneal Bevacizumab injection exhibited strong anti-tumor growth and anti-angiogenesis activity toward osteosarcoma in a nude mouse model. However, the authors claimed that Bevacizumab did not influence the incidence of mouse lung metastasis (109). As mentioned above, surgery combined with chemotherapy is still the first-line treatment for the advanced osteosarcoma. To date, in clinical trials, Bevacizumab has been used as an adjuvant therapy in combination with multiple chemotherapy drugs after surgery, if required. In 2017, Navid et al. reported the results of a phase II trial to evaluate the feasibility and efficacy of combining Bevacizumab with methotrexate, doxorubicin, and cisplatin (MAP) in patients with localized and resectable osteosarcoma (**Table 2**) (148). They claimed that the addition of Bevacizumab to MAP for osteosarcoma was tolerated, with low toxicity, except for frequent wound complications. However, the addition of Bevacizumab did not significantly improve the outcome of patients with localized osteosarcoma. In another clinical trial, Kuo et al. (149) reported that combining Bevacizumab with docetaxel and gemcitabine was well tolerated and had activity to treat relapsed or metastatic high-grade sarcomas (including eight patients with osteosarcoma) (**Table 2**). Collectively, the addition of Bevacizumab to multiple chemotherapy drugs to treat osteosarcoma induces no additional toxicity. However, the survival benefit is still unclear and large scale trials are needed.

R1507 is a monoclonal antibody recognizing the insulin-like growth factor-1 receptor (IGF-1R) (111), a receptor that plays an important role in tumor proliferation, apoptosis, angiogenesis, and metastasis (151). Thus, theoretically, R1507 should show some anti-angiogenic effect. In fact, R1507 has been proven to delay tumor growth in osteosarcoma mice xenograft tumors (152). A phase 2 trial also showed that R1507 is safe and well-tolerated in patients with osteosarcoma (150). However, the anti-angiogenic effect of R1507 in the clinic is still unclear. Despite the strong targeting and demonstrated efficacy in combination therapy for osteosarcoma, Mabs targeting VEGF or other proangiogenic factors face limitations in clinical application due to their high cost, uncertain survival benefit, and side effects (148–150). Therefore, extensive large scale clinical trials and more detailed research on the underlying molecular mechanism of Mabs targeting angiogenesis in osteosarcoma will be the potential research hotspots.

### 3.2 Tyrosine kinase inhibitors

TKIs are the most widely used angiogenesis inhibitors, being mainly used to block multiple signals downstream of VEGFR. Other targets include, PDGFR, Fms-like tyrosine kinase (FLT3), recombinant activated factor (RAF), and c-kit (a receptor tyrosine kinase, also called CD117 and stem cell factor receptor) (43). Research has shown that TKIs, such as Sorafenib, Sunitinib, Cediranib, Apatinib, Lenvatinib, Cabozantinib, and Regorafenib, show certain effects in the treatment of advanced osteosarcoma (**Table 2**). In particular, a phase 1/2 trial in 2021 showed that Lenvatinib combined with etoposide and ifosfamide could exert promising anti-tumor activity in patients with relapsed or refractory osteosarcoma; however, a high rate of treatment emergent adverse events was observed (119). The main concern related to TKIs is their poor organ targeting and short half-life inside the tumor, leading to limited efficacy and excessive side effects (119, 147). Treatment with TKIs causes secondary activation of the mTOR pathway in osteosarcoma (153). Small molecular inhibitors targeting the mTOR pathway, such as Everolimus, can effectively inhibit the proliferation and migration of ECs (153). Therefore, it was suggested that Everolimus could be used as a supplement to TKI therapy. However, clinicians should consider the adverse effects caused by mTOR inhibitors, such as high blood lipids, high blood sugar, mucositis, gastrointestinal reactions, and rashes, when formulating a treatment strategy (137).

### 3.3 Chinese herbal medicine

Chinese herbal medicine is believed to exert anti-angiogenesis effects via multiple targets and pathways, such as PI3K/AKT, Wnt/β-catenin, Janus kinase (JAK)/signal transducer and activator of transcription 3 (STAT3), Notch, NF-κB, and MAPK (154, 155). Moreover, many constituents of herbs have shown significant efficacy in anti-tumor and anti-angiogenesis therapeutics, with lower rates of adverse events (156, 157). For example, thymoquinone inhibits angiogenesis in osteosarcoma by inhibiting the NF-κB pathway (123). Triptolide can also inhibit angiogenesis in osteosarcoma through the HIF-1α, VEGF, and Wnt/β-Catenin pathways (124). Xie et al. reported that sinomenine, an active natural product derived from the plant *Sinomenium acutum Rehd. et Wils*, can effectively reduce CD147 and VEGF expression in OS cells through the C-X-C motif chemokine receptor 4 (CXCR4)-STAT3 pathway, thus inhibiting angiogenesis (125). Moreover, *Phyllanthus urinaria*, a widely used folk medicine in cancer treatment, could decrease the microvessel density and CD31 expression of osteosarcoma mouse xenografts, suggesting its potential anti-angiogenic effect (126). In addition, a recent study by Rabelo et al. reported that an extract of *Calotropis procera* could reduce angiogenesis and tumor progression in canine osteosarcoma cells by suppressing the expression of CD31, VEGF, osteopontin, and TGF-β (127). Collectively, these studies demonstrated that Chinese herbal medicine might be a promising adjunct treatment for advanced osteosarcoma via its anti-angiogenesis effects. However, to date, all the research on the use of herbal medicine in osteosarcoma has been carried out at using cellular and animal models and there remains a significant challenge to evaluate their safety in the clinic (157).

### 3.4 Potential metabolic antiangiogenic therapies in osteosarcoma

Osteosarcoma cells and ECs have similar metabolic characteristics, and regulating the metabolism of tumor cells mainly involves inhibiting nucleotide synthesis, inhibiting energy metabolism (e.g., glycolysis), and regulating redox metabolism and other metabolic pathways (158). These metabolic pathways also affect EC-guided angiogenesis in osteosarcoma (84). A study showed that 2-Deoxy-D-glucose (2-DG) could reduce osteosarcoma growth by inhibiting HK and lactate dehydrogenase A (LDHA), which are the key enzymes involved in anaerobic glycolysis (128). This signaling pathway also plays a key role in osteosarcoma angiogenesis. Bavachinin reduced osteosarcoma angiogenesis by inhibiting glycolysis via targeting HIF-1α and HK2 (129). Another study showed that Icariside II inhibits glucose metabolism and reduces HIF-1α-induced VEGF expression in human osteosarcoma cells, while simultaneously suppressing angiogenesis (130). Recently, Wang et al. reported that ANGPTL2 enhanced angiogenesis in osteosarcoma by upregulating the expression of HK2 and VEGF, and treatment with 2-DG could reversed this outcome (81) (**Table 1**). However, whether these drugs also act on similar metabolic targets in ECs and inhibit osteosarcoma angiogenesis requires further study.

### 3.5 Aptamers in osteosarcoma angiogenesis

An aptamer is an RNA or single stranded DNA with a specific three-dimensional structure, which acts as a starter or inhibitor trough that combines with specific molecules via Van der Waals forces or hydrogen bonds (159–161). Recent research has demonstrated that aptamers can act as tumor suppressors by impeding tumor angiogenesis, with advantages such as low immunogenicity, reversibility, wide target range, and short preparation cycle (162–164). Aptamers can be modified for different purposes. Modifications using fluorescein, biotin, and magnetic beads can amplify the transduction signals (165); adding polyethylene glycol (PEG) will increase their circulating time in the body; and binding them to drugs and NPs enable targeted delivery and controlled drug release (131, 162, 166–168). Liang et al. reported the construction of a specific aptamer (LC09) of osteosarcoma cells that was conjugated to a PEG-polyethylenimine (PEI) Cholesterol (PPC) lipopolymer, followed by combination with clustered regularly interspaced short palindromic repeats (CRISPR)/CRISPR associated protein 9 (Cas9) plasmids encoding *VEGFA* (131). They found that this system could inhibit osteosarcoma growth, lung metastasis, and angiogenesis *in vitro* and in osteosarcoma mouse models. However, further investigative studies are required to determine its clinical efficacy.

### 3.6 Nanoparticles in anti-vascular treatment

Nanotechnology is gradually being used in anti-vascular treatment of tumors, including osteosarcoma (131, 169). Utilizing flexible surface modification, researchers can promote drug targeting and prolong the half-life of nanotechnology-based drugs in tumors. Such NPs can release the drug precisely according to the specific microenvironment of the tumor, enhance efficacy, and reduce drug resistance and adverse reactions (170, 171). Nanotechnology can also overcome the shortcomings of traditional anti-tumor therapy and have a large capacity to combine anti-vascular therapy with chemotherapy and radiotherapy (170, 172). The development of nanopharmaceuticals has gone through three generations: The first generation was organ-targeted and aimed to increase the concentration of nanopharmaceuticals in solid tumors and reduce drug-induced off-target effects (173, 174). The second generation were cell-targeted, mainly focusing on surface modification of NPs to deliver drugs to specific tumor cells (175). The third generation are designed to deliver drugs to specific organelles, such as mitochondria, the endoplasmic reticulum, lysosomes, and nuclei (176–181).

In anti-vascularization therapy, many studies have focused on selectively delivering NP-loaded drugs to the tumor’s vascular system. Researchers modified short peptides on NPs to ensure their specific binding with integrin αvβ3 on the surface of tumor vascular ECs (182). Nano-particle platforms include liposomes, albumin NPs, polymer micelles, gold NPs, and mesoporous silica (183). Studies have placed effectors, such as small interfering RNAs (siRNAs) or other small molecule drugs (e.g., Rapamycin), on the surface of, or inside, NPs (169), thus allowing the successful delivery of the effectors into tumor ECs to exert a therapeutic function. Meanwhile, some studies focused on the combination of aptamers and NPs. As mentioned in the aptamers section, the osteosarcoma cell-targeted aptamer LC09 could be loaded into special NPs, which exert an anti-vascular effect with reduced adverse reactions (131). In addition, researchers assembled gold nanoclusters (GNCs) with Sgc8, an aptamer targeting TKIs, to form the GNCs@aptamer. This not only improved drug targeting, but also increased the duration of the drug’s residence in the tumor (184). Although there is still lack of research focusing on the anti-angiogenic effect of aptamers or NPs on osteosarcoma, this research direction might provide valuable new perspectives on anti-vascular treatment.

## 4 Challenges and perspectives

Multi-mode therapy, including surgery, chemotherapy, immunotherapy, radiotherapy, gene therapy, or other targeted drugs, can help to improve the therapeutic effect against osteosarcoma compared with any single method (185, 186). Anti-angiogenesis therapy is mainly used to inhibit the formation of new blood vessels, not to destroy existing vessels. Therefore, we still need to consider the coincidental destruction of existing blood vessels, because this might inhibit the delivery of drugs to the tumor. Traditional anti-angiogenic drugs, such as Everolimus, have many shortcomings in the treatment solid tumors, such as drug resistance, unexpected adverse reactions, poor targeting, and short tumor residency time.

In recent years, the role of metabolism in tumor anti-angiogenesis therapy has been widely recognized. Although limited research has been conducted on targeting EC metabolism in the anti-angiogenic therapy of osteosarcoma, recently it has attracted considerable attention (84, 91, 92). Consequently, we anticipate that metabolic regulation will emerge as a promising therapeutic strategy for advanced osteosarcoma in the future. However, several challenges still need to be addressed, including the identifying unique metabolic patterns specific to osteosarcoma, developing targeted drugs, and rigorously evaluating the safety and efficacy of metabolic regulation therapy. Therefore, further research and clinical exploration in related fields are imperative to propel the advancement of metabolic regulation in osteosarcoma treatment.

Additionally, the TME maintains the endothelium in a highly proliferative and metabolic state through various mechanisms. Studies have shown that the secondary hypoxia environment within the tumor after anti-angiogenesis treatment will stimulate a new pathway of angiogenesis, which might lead to drug resistance or tumor recurrence, and thus poor anti-tumor efficacy (187–189). Therefore, we anticipate that anti-angiogenic therapy targeting the TME will hold great promise for preventing drug resistance and tumor recurrence in the treatment of advanced osteosarcoma in the future.

Exploring new multi-targeted anti-vascular drugs or improving the pharmacokinetics of current drugs will also be helpful in future treatment strategies (190, 191). The ultimate aim is to induce a series of intracellular cascade reactions through multi-target synergies, exert a full range of anti-angiogenesis effects, maintain more stable pharmacodynamics, and avoid side effects and drug interactions (26, 192). In addition, the development of nanomedicine can help with the targeted delivery of drugs, improve their bio-availability, and reduce adverse reactions, thereby playing an innovative role in anti-tumor treatment (183, 193, 194). Comprehensive treatment based on nanotechnology can also combine chemotherapy, radiotherapy, anti-vascular therapy, and immunotherapy (170), thus exerting synergistic anti-tumor effects (105). However, many nanopharmaceuticals failed in phase II or III clinical trials, which was mainly ascribed to poor treatment effects (195). Therefore, while comprehensive treatment based on nanotechnology might be a potential strategy in future therapy, it requires improvement and optimization.

## 5 Conclusion

Angiogenesis is crucial for the growth, expansion, and metastasis of osteosarcoma. Therapy combining anti-angiogenic treatments and other methods has gradually emerged as the anti-tumor strategy with the most potential to treat advanced osteosarcoma. However, there are still some limitations that require optimization, such as an insufficient therapeutic effect, drug resistance, and side effects. Developing new angiogenesis inhibitors, exploring the possibility of multi-target drug combinations and sequential therapy, and combining classical drugs with NPs to improve delivery and reduce off-target side effects, will help to break the current bottleneck of treatment, thus providing more benefits to patients and improving their outcomes.
